# The Potential Role of Serum Tau Protein (MAPT), Neuronal Cell Adhesion Molecule (NrCAM) and Neprilysin (NEP) in Neurodegenerative Disorders Development in Psoriasis—Preliminary Results

**DOI:** 10.3390/jcm11175044

**Published:** 2022-08-27

**Authors:** Anna Baran, Julia Nowowiejska, Justyna Magdalena Hermanowicz, Beata Sieklucka, Julita Anna Krahel, Paulina Kiluk, Dariusz Pawlak, Iwona Flisiak

**Affiliations:** 1 Department of Dermatology and Venereology, Medical University of Bialystok, Zurawia 14 St., 15-540 Bialystok, Poland; 2 Department of Pharmacodynamics, Medical University of Bialystok, Mickiewicza 2C St., 15-540 Bialystok, Poland

**Keywords:** tau protein, neuronal cell adhesion molecule, neprilysin, MAPT, NrCAM, NEP, psoriasis, neurodegenerative disease, Alzheimer’s disease, Parkinson’s disease

## Abstract

Psoriasis is one of the most common dermatoses, which shortens patients’ lives because of the wide comorbidity. However, little is known about its association with neurodegenerative diseases (NDs). We aimed to investigate whether psoriatics are at increased risk of NDs. Sixty patients with plaque-type psoriasis were enrolled into the study. Serum concentrations of tau protein (MAPT), neuronal cell adhesion molecule (NrCAM) and neprilysin (NEP), which are NDs biomarkers and have been hardly studied in psoriasis before, were measured before and after 12 weeks of treatment with acitretin or methotrexate. NrCAM and NEP concentrations were significantly lower in patients than controls, whereas MAPT higher (all *p* < 0.05). There was no association between these markers and psoriasis severity, BMI or disease duration. After the treatment the concentration of NrCAM and NEP significantly increased and MAPT decreased (*p* < 0.001, *p* < 0.05, *p* < 0.01, respectively). Methotrexate had significant influence on the concentrations of all markers, hence it seems to have neuroprotective properties. Psoriasis severity and duration do not seem to affect the risk of neurodegenerative process. Our results suggest that NDs could be considered as another comorbidity of psoriasis and that further research are needed in order to establish their definite association.

## 1. Introduction

One of the most common diseases and sometimes the biggest therapeutic challenge in the dermatological practice remains psoriasis. It occurs with a frequency of 2–4% on average worldwide [1]. Its pathogenesis involves genetics, immunological disturbances, influence of environmental factors and chronic inflammatory condition [2]. In case of psoriasis, it is even called ‘metaflammation’ to emphasize the connection with metabolic disorders [3]. As for clinical picture, psoriasis presents with erythematous-papular lesions with scales, which may organize into psoriatic plaques [4]. However, it is not just a skin disease. Contemporary perception of psoriasis focuses on psoriasis as multi-organ disorder, accompanied by many comorbidities, which shorten patients’ life duration [5]. Psoriasis has been so far linked to different comorbidities, the issue of which tends nowadays to be included in psoriasis management guidelines. Probably the most known psoriasis comorbidities are arthritis and cardiometabolic complications [6,7], but there is also evidence of the association with ocular disorders, neoplasms, inflammatory bowel diseases and psychological burden [7,8]. That is why the search for new accompanying disorders and their markers is still required to properly manage psoriatic patients.

One such group of accompanying diseases are neurodegenerative diseases (NDs). They are defined as such in which loss of neuronal tissue occurs [9]. The most common NDs are for instance Alzheimer’s disease (AD), Parkinson’s disease (PD) or amyotrophic lateral sclerosis (ALS). Psoriasis and NDs share some pathogenetic background, especially genetic factors, oxidative stress, inflammatory pathways and metabolic disorders [10]. Several research studies have also indicated that psoriatics perform worse in different cognitive tests [11,12]. Currently, there are not many data on the potential association between psoriasis and NDs, the more on their biomarkers. Recently we published a paper on brain fatty acid-binding protein 7 (FABP), neurofilament light chain (NFL) and glutamic acid (GA) as potential indicators of increased NDs risk in psoriatics [13]. We found increased serum concentrations of FABP-7 and NFL in psoriatic patients (similar to what is observed in patients with NDs), which prompted us to search for other NDs markers in psoriatics [13]. This paper is the continuation of our research, where we present our study on three other proteins that are engaged in neurodegenerative processes: tau protein (microtubule associated protein tau, MAPT), neuronal cell adhesion molecule (NrCAM) and neprilysin (NEP).

MAPT aberration is probably one of the most famous hallmarks of AD. However, it is not specific for AD. There is a whole group of disorders that are characterized by the presence of abnormal MAPT aggregation, which are called tauopathies [14]. These include, besides AD, frontotemporal dementia with parkinsonism linked to chromosome 17 (FTDP-17) or Pick’s disease [14]. MAPT function is to stabilize microtubules formation [15]. It has been well-established that hyperphosphorylation of tau protein is involved in the pathogenesis of tauopathies [14,16]. Tau is an axonal protein, that under physiological conditions is phosphorylated to an optimal extent, which influences microtubules stability [15]. MAPT may form neurofibrillary tangles when it is truncated or hyperphosphorylated [16]. MAPT is 3–4 times more phosphorylated in the brains of patients with AD than in healthy persons; however, this process is not the only factor affecting filament aggregation and others are constantly investigated [15]. When the dementia symptoms of AD occur, blood-based tau concentrations are increased compared with subjects without cognitive impairment, although this observation is not as prominent as in cerebrospinal fluid (CSF) [17]. As for PD, the literature data are inconsistent, with some of them reporting increased and others similar serum concentrations to healthy controls [18,19]. Serum MAPT has also already been slightly investigated in psoriatic patients and turned out to be elevated [10].

NrCAM, according to its name, is an adhesion cell molecule from the L1 family of the immunoglobulin superfamily [20]. Its expression is observed in neurons and glia both in the central- and peripheral nervous systems [21]. It is involved in neuronal cells’ proliferation and differentiation, axons growth, synapses and myelin creation [20]. The role of NrCAM in the pathogenesis of different disorders has been established. These involve autism, glial cell tumors, melanoma, thyroid papillary carcinoma, colon cancer or susceptibility to addiction [20]. NrCAM is a physiological substrate for disintegrin and metalloprotease 10 (ADAM10), which is involved in amyloid precursor protein (APP) cleavage, so may exert a protective role in AD and therefore become a potential therapeutic target [22]. NrCAM has been proved to become a marker of selective ADAM10 activation in vivo [22]. NrCAM has been demonstrated to be decreased in patients with AD [22]. However, it has never been studied in psoriasis.

NEP is an enzyme considered among the family of proteases, which was the first discovered mammalian zinc-activated endopeptidase representative [23]. NEP is able to degrade insulin, metabolize neurotransmitters and convert endothelin [23,24]. Moreover, it has the same structure as encephalinase found in the brain and the marker of lymphocytes—CALLA [23]. Research data show that NEP is present in a variety of body organs, including brain, heart, lungs, thyroid and adrenal glands, as well as the gastrointestinal tract and kidneys [23]. NEP is probably most well-known in relation to its use in cardiology—NEP inhibitors are used in the treatment of heart failure [23], and cardiometabolic disorders (CMDs) are probably the best-confirmed comorbidities of psoriasis [25]. As for NDs development, it takes part in beta-amyloid cleavage, and amyloid accumulation is obviously a hallmark of AD [24]. It has been proved that NEP concentration is decreased in the brain of patients with AD and its increased activity is associated with lower amyloid concentrations, as well as exerts a protective function against neurotoxicity (in vitro) [24]. There is evidence that the measurement of NEP serum activity is decreased in patients with mild cognitive impairment and AD [26]. NEP has never been studied in psoriatic patients before.

Essentially, our study involved blood-based markers, so this method is relatively non-invasive, especially compared to CSF collection. Moreover, to the best of our knowledge, NrCAM and NEP have never been investigated in patients with psoriasis before, and MAPT had been studied only once before, so our research may shed some new light on the association between this dermatosis and the risk of NDs or on perhaps protective role of antipsoriatic therapy in that interplay.

## 2. Materials and Methods

60 patients (21 females and 39 males) with an exacerbation of plaque-type psoriasis, hospitalized at the Department of Dermatology, were enrolled into the study. Their median age was 57 (19–80) years old. They were compared with 30 sex- and age-matched volunteers without skin diseases. All participants signed informed written consents before initiation. None of the patients or controls were under any dietary restriction or were taking medications for at least three months before the enrollment. The exclusion criteria comprised other types of psoriasis, chronic inflammatory diseases and cardiometabolic, autoimmune or oncological comorbidities. Psoriasis area and severity index (PASI) was assessed by the same person in all patients. The investigated group was divided depending on the disease intensity into three sub-groups: mild (PASI 1) < 10 points, moderate (PASI 2) between 10 and 20, and severe (PASI 3) > 20. Body mass index (BMI) was calculated as weight/height^2^ (kg/m^2^). All subjects were further subdivided into groups according to BMI: BMI 1 was related to normal-weight (BMI 18.5–24.9) and included 12 persons; the second group—BMI 2—overweight (BMI 25–29.9) was noted in 13 psoriatic patients; and BMI 3, obesity (BMI > 30) was observed in 10 patients. Laboratory tests including C-reactive protein (CRP), complete blood count (CBC), serum glucose, total cholesterol (Chol), HDL, LDL, triacylglycerol (TGs) and transaminases (AST, ALT) were performed before treatment. The patients received two systemic treatment options: 15 persons took methotrexate (MTX) 15 mg/week using folic acid supplementation (15 mg/week, 24 h after MTX intake) and 20 subjects were started on acitretin at a dose 0.5 mg/kg/day. The treatment period lasted 12 weeks. The study was approved by the Bioethical Committee of Medical University in Bialystok (number: R-I-002/429/2017) and was in accordance with the principle of the Helsinki Declaration. 

### 2.1. Serum Collection 

Fasting blood samples were received from healthy subjects and patients before and after 12 weeks of treatment using vacutainer tubes with a clot activator. Samples were centrifuged at 2000× g for 10 min and preserved at −80 °C until analyses. MAPT, NrCAM and NEP levels were measured using an enzyme immunoassay kit supplied by Cloud Clone^®^ (SEB983Hu, SEC668Hu, SEB785Hu). Optical density was read at a wavelength of 450 nm. The concentrations were measured by interpolation from calibration curves prepared with standard samples supplied by the manufacturer. All the tests were performed by the same investigator in standardized laboratory settings. 

### 2.2. Statistical Analysis 

Normality of distribution was tested using the Shapiro–Wilk W test. The normally distributed data were analyzed using a one-way analysis of variance (ANOVA) and shown as mean SD. The non-Gaussian data were presented as median (full range) and analyzed using the non-parametric Kruskal–Wallis test. The Student’s t-test or nonparametric Mann–Whitney test were used to compare differences between the psoriasis group and the control group. The correlations were analyzed using Spearman’s Rank correlation analysis. Statistical analysis was conducted using the GraphPad Prism 9.20 software (GraphPad Prism 9.20 Software, La Jolla, San Diego, CA, USA). The differences were deemed statistically significant when *p* < 0.05.

## 3. Results

The prospective study included 60 patients with exacerbation of plaque-type psoriasis (study group), 21 women and 39 men, with the median age of 57 (19–85) years old and 30 individuals without dermatoses (control group) of the median age of 52.5 (25–64) years old, matched for age and BMI. The median value of BMI of the patients was 27.1 (17–44.4) kg/m^2^. Severity of psoriasis expressed by median PASI score was 15.4 before treatment and 6.95 after therapy. Baseline characteristic of the control and patients groups are summarized in Table 1.

### 3.1. MAPT

**Figure 1:**
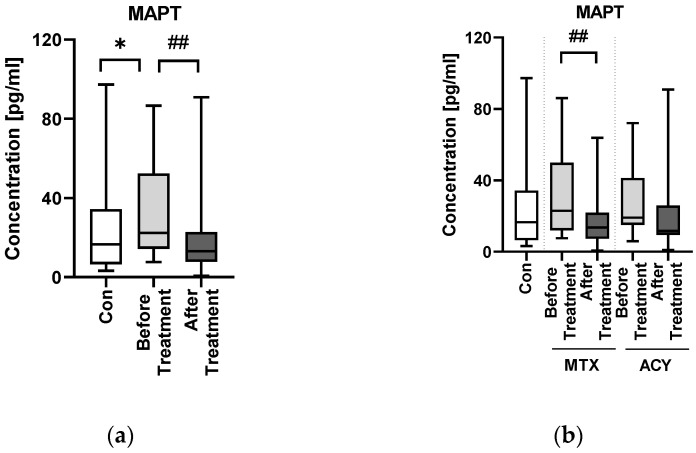

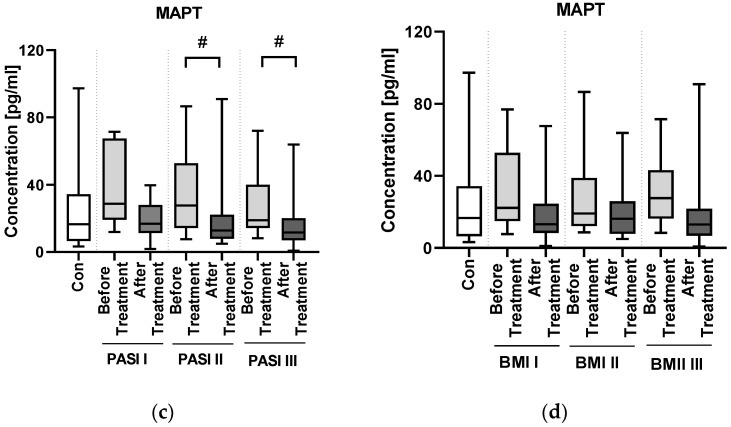
The levels of MAPT in psoriatics before and after total treatment (**a**), both drugs separately (**b**) and with regard to PASI (**c**) and BMI (**d**) subdivision compared to controls. * means the existence of statistically significant difference between patients single group compared to controls with *p* < 0.05; #/## means the existence of statistically significant difference between patients before and after the treatment with *p* < 0.05/<0.01.

The median serum MAPT concentration in patients with psoriasis was 22.35 pg/mL before treatment and was significantly higher compared to the controls: 16.55 pg/mL (*p* < 0.05) (Figure 1a). 

PASI subdivision did not reveal any significant differences before total therapy; however, in PASI II and PASI III subgroups, MAPT level decreased significantly afterward (both *p* < 0.05) (Figure 1c). MAPT did not differ between the BMI subgroups and compared to the controls (Figure 1d).

Serum MAPT level did not correlate with psoriasis severity expressed with PASI score, nor BMI, both before and after therapy (all *p* > 0.05) (Table 2). 

Analyzing possible associations between tau protein and demographic, clinical or laboratory parameters of the patients, including inter alia metabolic or inflammatory indices such as CRP, WBC, glucose level, lipids profile and BMI, no statistically significant relations were noted (all *p* > 0.05) (Table 3). 

**Figure 2:**
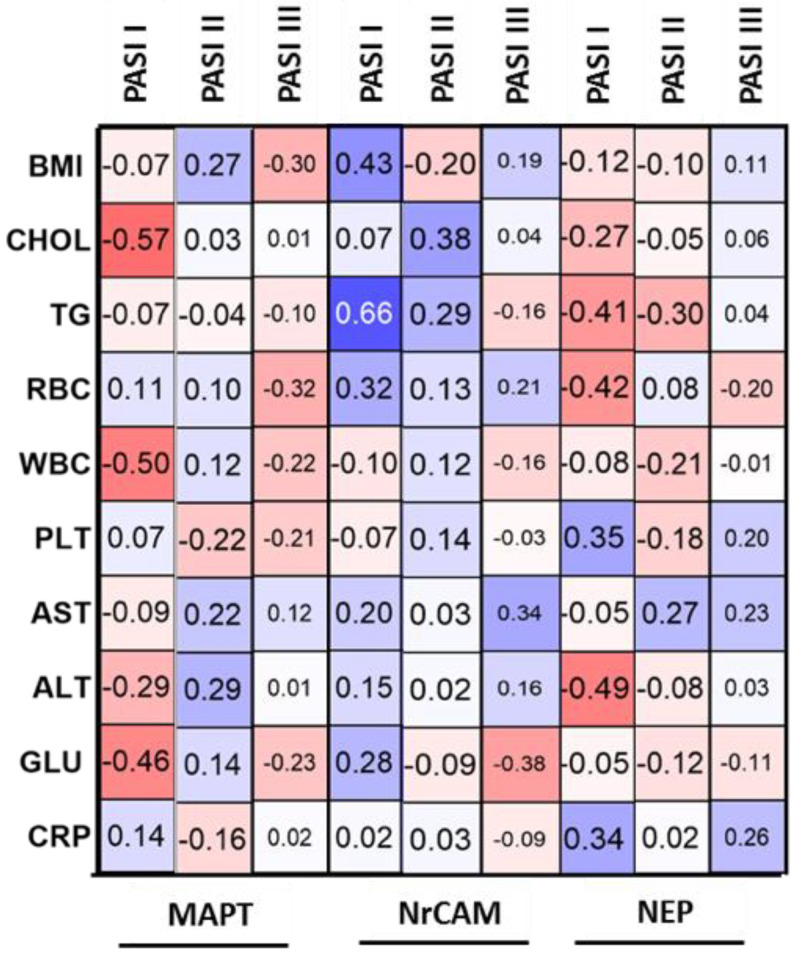
Correlations between evaluated proteins and selected laboratory parameters with regard to PASI subdivision before total treatment with use of Spearman’s rank correlation.

However, subdivision according to BMI or PASI revealed some significant relations (Figure 2). 

**Figure 3:**
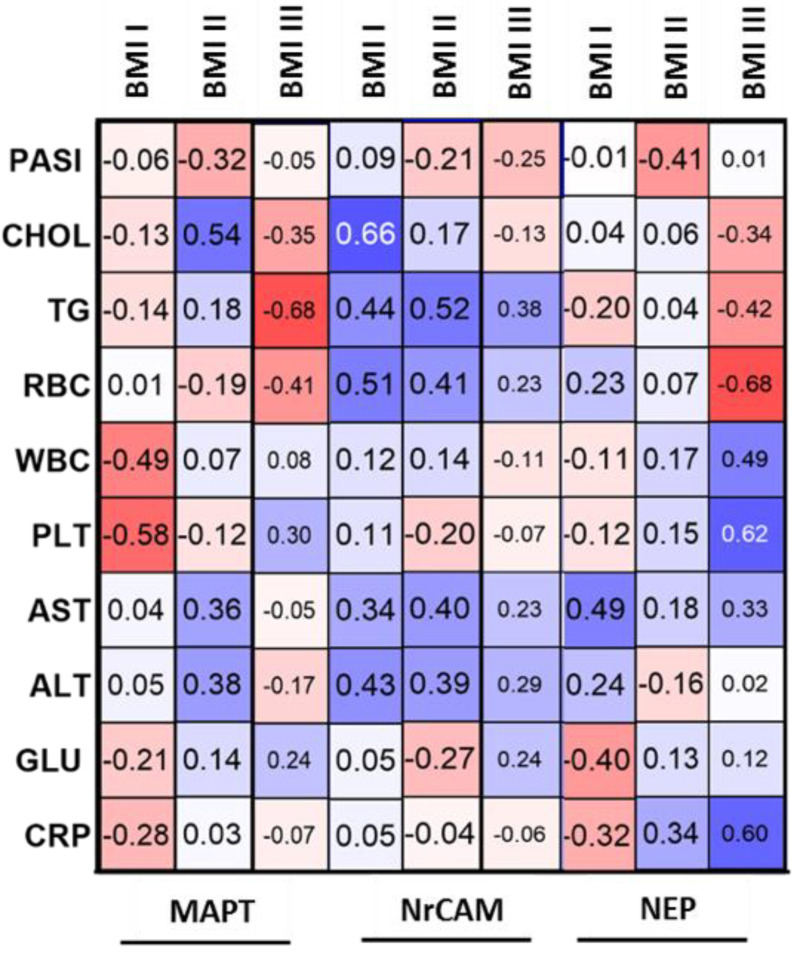
Correlations between evaluated proteins and selected laboratory parameters with regard to BMI subdivision before total treatment with use of Spearman’s rank correlation.

In patients with mild psoriasis, MAPT correlated negatively with total cholesterol, WBC and glucose levels (Figure 2). Further, in patients of normal weight significant negative correlations between the MAPT and WBC or PLT were noted (Figure 3). In overweight psoriatics MAPT was significantly positively correlated with total cholesterol concentration and in obese—negatively with TG (Figure 3).

**Figure 4:**
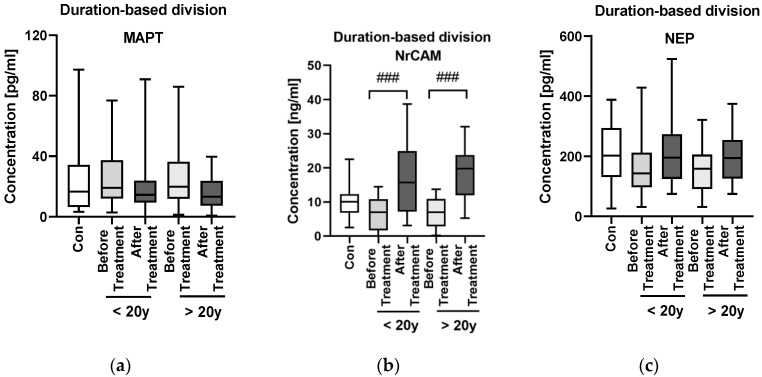
The levels of MAPT (**a**), NrCAM (**b**) and NEP (**c**) in psoriatics before and after total treatment compared to controls depending on the duration of psoriasis. ### means the existence of statistically significant difference between patients before and after the treatment with *p* < 0.001.

The duration-based division of the patients group with a threshold of 20 years showed that the duration of psoriasis does not influence significantly MAPT level (Figure 4a). 

After twelve weeks of systemic therapy a significant clinical improvement reflected inter alia by PASI decrease was observed (*p* < 0.001). The median MAPT serum level decreased significantly to 13.16 pg/mL after the therapy (*p* < 0.01), approaching the level similar as in the controls (Figure 1a). After division into subgroups of patients treated with the drugs separately, methotrexate in contrast to acitretin resulted in significant decrease in MAPT level (*p* < 0.01) (Figure 1b).

### 3.2. NrCAM

**Figure 5:**
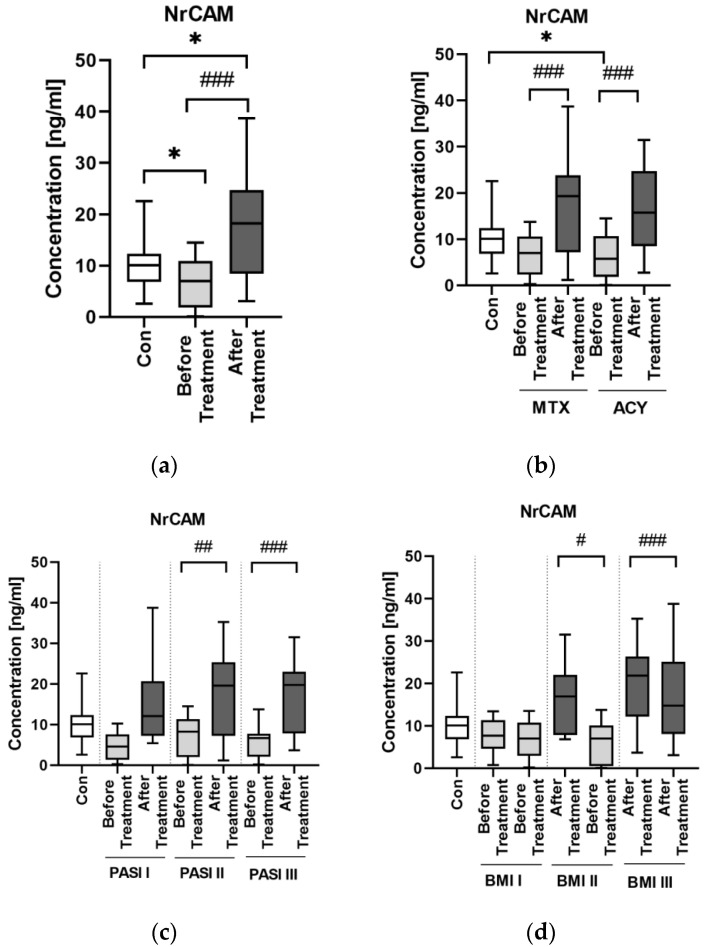
Serum NrCAM in psoriasis patients before total therapy (**a**), both drugs separately (**b**) and with regard to PASI (**c**) and BMI (**d**) subdivision. *—means the existence of statistically significant difference between patients single group compared to controls with *p* < 0.05; #/##/###—means the existence of statistically significant difference between patients before and after the treatment with *p* < 0.05/*p* < 0.01/*p* < 0.001.

Median serum NrCAM level was significantly decreased in patients with psoriasis compared to the controls (*p* < 0.05) (Figure 5a). 

The protein did not correlate with PASI score before therapy (*p* > 0.05). After division of the study group, we found that NrCAM concentration significantly increased after total therapy (*p* < 0.001) and was significantly higher than in the controls (*p* < 0.05) (Figure 5a). In BMI subgroups the median NrCAM level was the highest in obese psoriatics and it was higher than in the controls (*p* > 0.05) (Figure 5d). After the division basing on the duration of psoriasis, no significant difference in NrCAM concentrations was noted beside its significant increase after the treatment in both subgroups (Figure 4b).

With regard to BMI, no correlation was observed both before and after therapy (*p* = 0.07, *p* = 0.75, respectively) (Table 2). However, in patients with normal weight and obesity NrCAM positively correlated with BMI before therapy which changed afterward. Regarding relations with laboratory parameters, NrCAM correlated negatively only with ALT (R = −0.44) (Table 3). However, inside PASI subgroups some relations were noted. In PASI I subgroup we observed a positive correlation of NrCAM with TG concentration (R = 0.66) (Figure 2). In patients with moderate-to-severe psoriasis there was a positive trend for total cholesterol concentration (R = 0.38) (Figure 2). In normal weight psoriatics, NrCAM positively correlated with total cholesterol concentration, TG, RBC and ALT activity (R = 0.66, R = 0.44, R = 0.51, R = 0.43, respectively) (Figure 3). In overweight patients the protein positively correlated with TG, RBC and AST (R = 0.52, R = 0.41, R = 0.4, respectively) (Figure 3).

After the treatment median serum NrCAM concentration significantly increased (*p* < 0.001) (Figure 4a,b). After division according to a specific drug, a significant increase was noted both for methotrexate and acitretin (both *p* < 0.001). The increase in NrCAM after the treatment was noted especially in PASI II and PASI III subgroups (both *p* < 0.001) (Figure 4b). 

### 3.3. NEP

**Figure 6:**
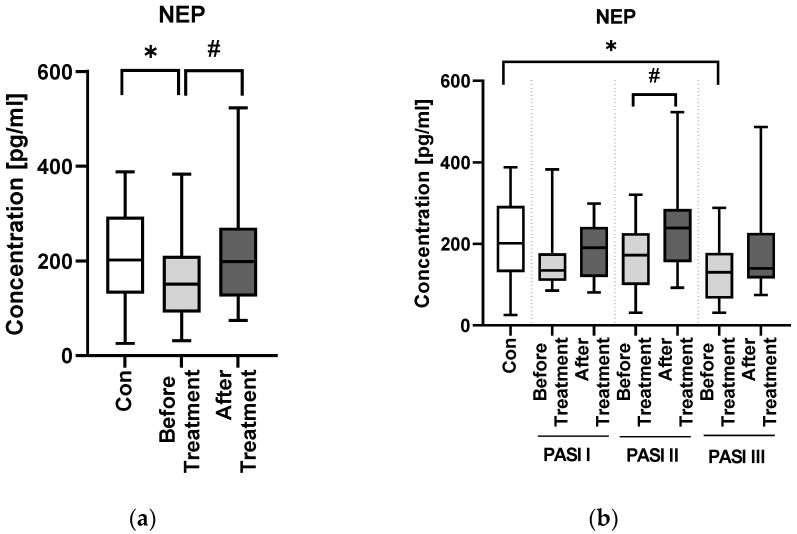

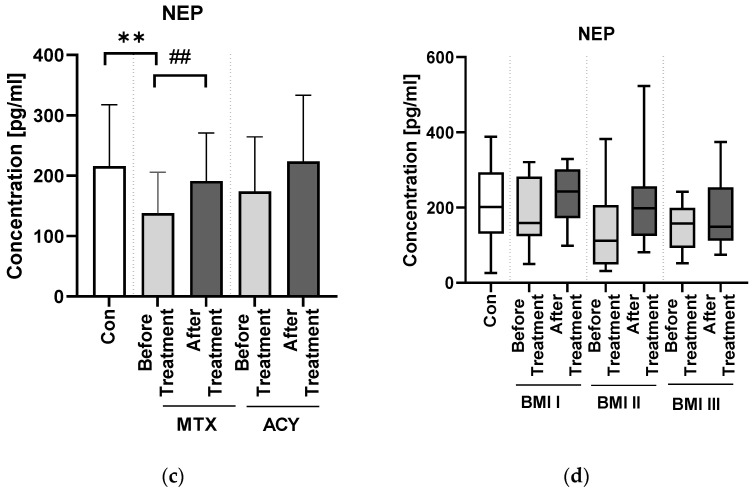
Serum NEP in psoriasis patients before and after total therapy (**a**), both drugs separately (**b**) and with regard to PASI subdivision (**c**) and BMI (**d**). */** means the existence of statistically significant difference between patients single group compared to controls with *p* < 0.05/*p* < 0.01; #/## means the existence of statistically significant difference between patients before and after the treatment with *p* < 0.05/*p* < 0.01.

Median serum NEP concentration in patients was significantly lower than in the controls (*p* < 0.05) (Figure 6a).

Inside the PASI and BMI subgroups, NEP levels did not differ significantly (Figure 6b,d). Regarding possible relations of NEP with laboratory parameters no significant ones were found (Table 3). However, in mild psoriasis the protein correlated negatively with TG, RBC and ALT activity (R = −0.41, R = −0.42, R = −0.49, respectively) (Figure 2). According to BMI subgroups, in normal weight psoriatics NEP was related positively with AST activity (R = 0.49) and negatively with glucose level (R = −0.4) (Figure 3). In overweight patients a negative correlation between the protein and PASI was noted (R = −0.41) (Figure 3). Finally, in obese ones NEP correlated positively with WBC, PLT and CRP levels (R = 0.49, R = 0.62, R = 0.6, respectively) and negatively with TG or RBC (R = −0.42, R = −0.68, respectively) (Figure 3). The psoriasis duration had no important impact on serum NEP concentrations also with relation to the therapy (Figure 4c).

After the treatment, the median NEP level increased significantly with achieving the level as in the control group (Figure 6a). These relations were especially visible in patients with moderate-to-severe psoriasis (Figure 6b). With regard to certain drug influence, methotrexate was the one in contrast to acitretin which resulted in a significant increase in serum NEP level (*p* < 0.01) (Figure 6c).

## 4. Discussion

This research is a continuation of our previous study attempting to establish the possible relations between NDSs and psoriasis in order to evaluate whether psoriatics are at greater risk of cognitive impairment. Similar to our other paper, this one also seems to suggest that there could actually be a susceptibility to NDs among psoriatics, based on their blood biomarkers. We managed to find statistical significance in evaluated NDs biomarkers serum concentrations between psoriatics and subjects without psoriasis, moreover in all cases—also a significant difference in their concentrations after the antipsoriatic treatment. It surely gives new insight into the interplay between psoriasis and NDs and indicates the need for exploring this issue.

MAPT aberrations have been observed in different neurological conditions [14]. As for the usefulness of blood as a biological fluid for concentration measurement, it has been established that MATP is elevated in the blood of patients with AD and in some cases also in PD [19]. In PD it was discovered to positively correlate with cognitive performance and hippocampal atrophy [18,19]. Previous research also suggested that the increased blood level of total MAPT was associated with a faster progression of dementia [17]. In our study, MAPT concentration was significantly higher in patients than in controls—the same observation that has been made also in some NDs. In the only paper in which MAPT had been studied in psoriatics before, the same results were obtained [10]. Such observations may indicate that the probability of dementia and NDs occurrence in psoriatics may be increased and surely requires more in-depth research to be elucidated.

Psoriasis severity expressed by PASI, as well as BMI and duration of psoriasis do not seem to affect MAPT concentration and therefore the risk of NDs. There was no direct correlation between PASI and MAPT concentration, as well as after division into subgroups according to psoriasis severity nor BMI. These observations were also made in our previous paper regarding three other ND biomarkers, which even more confirms our hypothesis. Apparently, psoriasis severity or duration do not influence MAPT concentrations and thus do not directly indicate an increased risk of NDs. The different outcome was presented in the paper by Okan et al. who reported that serum MAPT concentration may be correlated with PASI in patients with psoriasis and that the age of psoriasis onset before 40 years old affects higher MAPT concentrations [10], although this study was performed on a group relatively smaller than ours. Therefore, we conclude that PASI and psoriasis duration cannot be perceived as prognostic factors of NDs development possibility at this moment. It has to be verified in further studies.

We did not find any correlations between MAPT concentration and basic laboratory parameters in relation to the group of patients as a whole. However, we observed a negative correlation between MAPT and glucose, WBC and cholesterol levels in patients with mild psoriasis, therefore we might suspect that it could indicate its protective influence against the metaflammation in such individuals.

The extent of tau pathology depends on the dose, hence the idea for tau-lowering therapy. Another way is inhibition of tau aggregation and tau phosphorylation [27]. In our study, after the antipsoriatic treatment MAPT concentration significantly decreased. After division into two subgroups according to the administered systemic agent, it was methotrexate which caused the significant drop in tau protein concentration. Therefore, basing on this observation, we could conclude that methotrexate could be considered as the drug of choice in patients with elevated MAPT serum concentration. Further, presumably methotrexate might exert protective effect via downregulation of tau protein on cognitive impairment in psoriatic patients. However, of note, the literature data are conflicting. One study by Judge et al. mentions that methotrexate may exert a protective role on cognitive function in patients with rheumatic diseases [28], which is consistent with our results. On the other hand, the paper by Elens et al. seems to state the opposite: they indicate that methotrexate may contribute to the increased MAPT hyperphosphorylation. Methotrexate is among others administered to children with leukemia in order to provide CNS prophylaxis and apparently this agent, which is the dihydrofolate reductase inhibitor, alters the distribution of intracellular folates, decreases the CSF folates and increases MAPT concentrations [29]. In the paper on mice model by Elens et al. they observed that changes in 5- methyltetrahydrofolate serum concentrations and in CSF are associated with increased MAPT concentrations in CSF, which leads to decreased cell proliferation in the hippocampus [29]. This observation seems to be age-dependent though and cited study was performed on mice. Obviously, this issue requires further treatment, but based on available data and our study methotrexate seems to exert a protective effect on cognitive impairment. As for the possible influence of acitretin on MAPT, unfortunately we did not find any information. Similarly, we did not manage to find any report on the potential influence of tau phosphorylation inhibitors, used in AD treatment, on psoriasis. Definitely, the interrelations between both methotrexate and acitretin should be further explored.

NrCAM is a marker of ADAM10 activity and in case of its decreased activity, which may be monitored by decreased concentration of NrCAM, APP is less degraded and therefore more beta-amyloid accumulates, which is an important link in AD pathogenesis [22]. In our study, the concentration of NrCAM was significantly lower in patients before the treatment than in controls—exactly as in patients with AD, which may be another potential proof for their association. Similar to MAPT, NrCAM concentration was not associated with psoriasis severity or duration. Although there was no direct correlation between NrCAM and BMI, the highest concentrations were noted in obese patients which might point to links with adipose tissue or perhaps chronic metaflammation increasing within the weight observed in psoriasis.

We did not find any correlations between NrCAM concentration and basic laboratory parameters in relation to the group of patients as a whole, except for the negative correlation with ALT activity. Apparently, NrCAM might be associated with liver dysfunction in psoriatic patients, which needs to be further elucidated.

After the treatment NrCAM concentration increased significantly. Both systemic antipsoriatic drugs caused a significant increase in NrCAM concentration. Acitretin has been described to enhance the activity of ADAM10 and to have some kind of sparing effect on NrCAM, thus our results remain in accordance with the literature data stating that this drug should increase NrCAM concentration [22]. This is why acitretin, which is widely used for systemic antipsoriatic therapy could be also used in AD therapy. As for methotrexate, it caused a significant elevation of NrCAM, but we did not find any information on its possible influence on the protein in the available medical literature so the discussion at this point is inconclusive.

Under the natural circumstances in healthy persons, amyloid formation and clearance are balanced, therefore there is no unfavorable accumulation observed [30]. The problem occurs when formation excesses the clearance and that could be associated with decreased activity of enzymes involved, of which the most important is NEP [30]. In our study NEP concentration was significantly lower in patients than in controls—such observation has been made also in NDs—hence again another cognitive impairment biomarker tends to be beyond normal limits in psoriatic patients.

Psoriasis severity probably does not have an important impact on serum NEP concentration. However, we also found that in overweight patients PASI was negatively correlated with NEP, which could mean that patients with elevated body weight and more severe psoriasis are more prone to cognitive impairment. Hence, we believe it could suggest that psoriasis severity does not directly affect the risk of neurodegeneration but it may be important in patients with severe cases of psoriasis associated with obesity—perhaps such subjects are more susceptible to NDs development. This is what we managed to observe in our previous research, and it is consistent with the data indicating greater psoriasis severity in patients with higher body mass.

We did not find any correlations between NEP concentration and basic laboratory parameters in relation to the group of patients as a whole. However, we would like to point out that in obese psoriatics, NEP negatively correlated with TG. This observation reflects the literature data indicating the relationship of dementia with metabolic disorders, including obesity and dyslipidemia, which may exacerbate cognitive impairment [10]. 

NEP has been suggested not only as a marker of cognitive impairment but also as a possible tool for its monitoring and treatment efficacy assessment [26]. In our study, NEP concentration significantly increased after the antipsoriatic treatment, so we may assume that such therapy may have a beneficial influence on cognitive function and decrease the risk of such complications. When analyzing particular antipsoriatic agents, we noticed that both of them caused the elevation in NEP concentration, but methotrexate significantly. Therefore, we may suspect that this drug could be the therapy of choice in patients with decreased NEP concentrations but also perhaps of protective effect on NDs development. As for the literature data on the possible influence of methotrexate and acitretin on NEP, we did not manage to find any information. As NEP inhibitors are approved as drugs used in heart failure therapy, it could raise the question of whether they could cause cognitive impairment. Apparently, there is no evidence to state that, but further, longer observations are needed [23,30]. At this moment we would like to highlight the tight association between psoriasis and cardiometabolic syndrome [5], which makes it even more possible that psoriatics would be prescribed such drugs, so the observation of such patients towards ND complications may turn out to be necessary. There were also preclinical studies in which *NEP* gene was transferred to mice overexpressing human amyloid precursor protein and successfully lead to decreased amyloid accumulation [30]. This is a promising idea; nevertheless, there are no such drugs available so far.

Our study is one of the first of its kind, which surely brings some novelty into the field of psoriasis, but on the other hand, the obtained results may be difficult to analyze since we do not have other studies on psoriatics to compare with. As for pointing out the limitation of our study, patients and controls came from the same single department. They were all from the same geographical location and Caucasian ethnicity. We investigated only blood serum biomarkers without measurement in CSF or tissue samples. In the future, we plan to expand our study group and methods in cooperation with neurologists or psychiatrists. We are aware that as for now our results should be treated as preliminary but still encouraging to develop this issue.

## 5. Conclusions

Psoriasis is one of the most common skin diseases and the cause of medical, social and economic burden worldwide. Its comorbidities have been so far thoroughly investigated, but still, there is much to find out in order to properly manage psoriatic patients. Based on our results we suggest that it should be potentially considered that psoriasis may predispose to NDs development. Moreover, psoriasis severity and duration probably do not affect the risk of NDs development, apart from in obese patients and with severe skin lesions. Such conclusions are the more possible since we obtained similar results in our previous study, where we investigated three different NDs biomarkers. MAPT seems to exert a protective influence on metaflammation. NrCAM may be associated with liver function in psoriatics. Methotrexate could be perhaps considered in patients with low NEP and high MAPT concentrations. In the case of decreased NrCAM, both methotrexate and acitretin seem to be equally effective. Based on previous others and our studies, methotrexate emerges to be also considered a ‘neuroprotective’ agent. Nevertheless, more studies are required to establish the real association between psoriasis and NDs.

## Figures and Tables

**Table 1:** Baseline characteristics of patients and controls.

	Controls (n = 30)	Patients (n = 60)
Sex [M/F]	10/20	39/21
Age [years]	52.5 (25–64)	57 (19–85) NS
Height [cm]	166 (156–186)	176 (154–190) NS
Weight [kg]	67.5 (50–133)	(83 (54–136) NS
BMI [kg/m^2^]	24.6 (20–41)	27.1 (17–44.4) NS

NS, non-significant.

**Table 2:** Correlations of the proteins with PASI and BMI before and after total therapy.

Protein	PASI		BMI	
	Before Therapy (R, *p*)	After Therapy (R, *p*)	Before Therapy (R, *p*)	After Therapy (R, *p*)
MAPT	−0.149; 0.316	0.241; 0.094	−0.015; 0.919	−0.103; 0.478
NrCAM	−0.059; 0.736	−0.275; 0.090	−0.141; 0.419	−0.083; 0.617
NEP	−0.011; 0.932	−0.142; 0.284	−0.062; 0.652	−0.212; 0.109

**Table 3:** Correlations between the proteins and selected laboratory parameters before therapy.

	AST R; *p* Value	ALT R; *p* Value	GLU R; *p* Value	CRP R; *p* Value	Chol R; *p* Value	TG R; *p* Value	Rbc R; *p* Value	WBC R; *p* Value	PLT R; *p* Value
MAPT	0.13; 0.38	0.9; 0.55	−0.03; 0.863	−0.05 0.727	−0.02 0.884	−0.07 0.661	−0.1 0.496	−0.14 0.348	−0.21 0.153
NrCAM	−0.12; 0.497	−0.44; 0.008 **	−0.18; 0.293	0.12; 0.493	0.01; 0.938	−0.20; 0.242	−0.32; 0.059	−0.01; 0.961	0.08; 0.663
NEP	0.21 0.134	−0.11 0.446	−0.08 0.553	0.13 0.349	−0.05 0.712	−0.19 0.177	−0.12 0.388	0.05 0.701	0.05 0.717

**—means the existence of statistically significant difference between patients single group compared to controls with *p* < 0.01.

## Data Availability

Data available on the request.
